# Supplementary material for: RNA polymerase II stalling at pre-mRNA splice sites is enforced by ubiquitination of the catalytic subunit
Source: eLife. 2017 Oct 13;6:e27082. doi: 10.7554/eLife.27082 (PMC5673307; doi:10.7554/eLife.27082)
Supplement: Supplementary file 1. [file elife-27082-supp1.docx]

**Table S1**

| Dataset | Number of mapped reads |
| --- | --- |
| Bre5-HTP [rep 1] | 119640 |
| Bre5-HTP [rep 2] | 79064 |
| Bre5-HTP, ubp3∆ [rep1] | 32112 |
| Bre5-HTP, ubp3∆ [rep2] | 60677 |
| Bre5-HTP, 37°C | 130148 |
| Bre5-HTP, rps5-3 | 268759 |
| Bre5-HTP, rps5-3 at 37°C | 167617 |
| BY4741 (untagged strain) | 6823 |
| Rpo21-HTP total | 2326314 |
| Rpo21-HTP, bre5∆ total | 1373037 |
| Ub-Rpo21-HTP | 2349370 |
| Rpo21-HTP, bre5∆ GST | 315011 |
| Rpo21-HTP GST | 262826 |
| Ub-Rpo21-HTP bre5∆ | 1749177 |

Table S1: Number of mapped reads in the CRAC datasets used in this study. PCR duplicates have been removed.

**Table S2**

| Dataset | EI | IE | EE | 2EE/(EI+IE) |
| --- | --- | --- | --- | --- |
| Rpo21-HTP | 9232 | 4346 | 1208 | 0.18 |
| Bre5-HTP [rep 1] | 12 | 19 | 530 | 34.19 |
| Bre5-HTP [rep 2] | 1 | 18 | 290 | 30.53 |
| Bre5-HTP, ubp3∆ [rep1] | 0 | 3 | 298 | 198.67 |
| Bre5-HTP, ubp3∆ [rep2] | 0 | 1 | 544 | 1088.00 |
| Bre5-HTP, 37°C | 12 | 21 | 656 | 39.76 |
| Bre5-HTP, rps5-3 | 42 | 112 | 1803 | 23.42 |
| Bre5-HTP, rps5-3, 37°C | 18 | 48 | 644 | 19.52 |

Table S2: The relative recovery of spliced mRNA versus unspliced pre-mRNA is expressed as the ratio of reads spanning exon-exon (EE) to exon-intron (EI) plus intron-exon (IE) junctions (2EE/(EI+IE)). The number of reads mapped to the different junctions and the spliced to unspliced ratio are indicated.

**Table S3**

| Strain | Genotype | Reference or source |
| --- | --- | --- |
| BY4741 | *MATa, his3∆, leu2∆, met15∆, ura3∆* | Brachmann, C. B. et al Yeast 1998 |
| YLM468 | BY4741, bre5::NAT | This study. |
| YLM473 | BY4741, ubp3::KAN | This study |
| YLM478 | BY4741, *Bre5HTP::URA* | This study. |
| YLM502 | YLM478 but ubp3::KAN (1) | This study. |
| YLM503 | YLM478 but ubp3::KAN (2) | This study. |
| YLM504 | BY4741, Rpb3TAP::HIS3 | This study. |
| YLM535 | BY4741, trp1::URA3 (1) | This study. |
| YLM557 | YLM504 but trp1::URA3 | This study. |
| YLM563 | YLM557 but bre5::NAT | This study. |
| YLM567 | YLM557 but ubp3::KAN | This study. |
| YLM594 | BY4741 Rpo21HTP::URA3 | Milligan et al. 2016 |
| YLM598 | YLM594 but bre5::NAT | This study. |
| YLM600 | RSP5 but Bre5HTP::URA3 | Neumann S. et al and this study. |
| YLM604 | Rsp5-3 but BRE5HTP::URA3 | Neumann S. et al and this study. |
| YIK91/Ribo1 |  | Alexander R. D. et al 2010. |
| YIK91/5’SSRibo1 |  | Alexander R. D. et al 2010. |
| YLM611 | YIK91/Ribo1 but bre5::KAN | This study. |
| YLM624 | YIK91/Ribo1 but ubr2::KAN | This study. |
| YLM681 | YIK91/5’SSRibo1 but bre5::KAN | This study. |
| YLM688 | BY4741 Rpo21K1246R HTP::URA | This study. |
| YLM512 | BY4741 Bre5F453A HTP::URA3 | This study. |
| YLM513 | BY4741 Bre5F421A F453A HTP::URA3 | This study. |
| YLM516 | BY4741 Bre5F421A R423E F453A HTP::URA3 | This study. |
| YLM518 | BY4741 Bre5K445E HTP::URA3 | This study. |

Table S3: Strains used in this study.

**Table S4**

| Name | Sequence |
| --- | --- |
| Bre5 F1 | CACTAATAGTTGCATTTGAAGTCATACCCTCGAATAGAAGTATCAAATAAAAGAAACGGATCCCCGGGTTAATTAA |
| Bre5 R1 | CTCATTTAAATGGCATTCATTCCCTTCCGCGCCAGTATATTAGTTACTAATCATACAGGAATTCGAGCTCGTTTAAAC |
| Bre5HTP for | CATATTTACTAATGGAACACGTTCTCATAGAAAGCAACCCCTAAAAAGAAAGGACGAGCACCATCACCATCACC |
| Bre5HTP rev | CTTTTTCAAATTTTTTTATTATTTTTTCAATTTTTCTTTTTAAAAGGCTTGTGGTTGATACGACTCACTATAGGG |
| \| Bre5HTPF453A \| \| --- \| | GGATAACAGCTGCAGACAATGCTGCCGTCGTCGACTTTGA |
| Bre5HTPK485E F | TTGAAGTATGCTTAGAAAGAGAAACAGTAAAGAAGCCCACGA |
| Bre5HTPY421A F | AGGACGGCTTTTATCCAATCGCCATTAGAGGTACAAATGG |
| Bre5HTPY421A_R423E F | AGGACGGCTTTTATCCAATCGCCATTGAAGGTACAAATGG |
| Ubp3 F1 | GACTCGTCTGCTACCATCATCCAGGTACCGCTTTCCTTTCCATCATCATTAAAAAAACGGATCCCCGGGTTAATTAA |
| Ubp3R1 | GTATTATTGCTATATTATTTTTTATGTATTTTGTCTATAATACCACCCCCCGTCGAATTCGAGCTCGTTTAAAC |
| Ubr2 F1 | CTGATAGTAAAGGTAAGATTCGTTAACTAAATTAATAGCTACTTAACAAGCACGCCGGATCCCCGGGTTAATTAA |
| Ubr2 R1 | CGGATACGCTAATTTCGTAGCAATTTTGAATGACTAGACATTTGTTGGATAAGAATTCGAGCTCGTTTAAAC |
| Rpb3 F2 | AAACCCCTTAATTGTTGTCATGAGGAAAATAACCAACAAAGTGGGCGGTTTCTTTGCAGACGGATCCCCGGGTTAATTAA |
| Rpb3 R1 | CTTATTATTTTCGGTTCGTTCACTTGTTTTTTTTCCTCTATTACGCCCACTTGAGAAGAATTCGAGCTCGTTTAAAC |
| Trp1 F1 | GACTATTGAGCACGTGAGTATACGTGATTAAGCACACAAAGGCAGCTTGGAGTCGGATCCCCGGGTTAATTAA |
| Trp1 R1 | GGCCTGCAGGCAAGTGCACAAACAATACTTAAATAAATACTACTCAGTAATAACGAATTCGAGCTCGTTTAAAC |
| Rpo21HTP For | CTCCTGCATATTCTCCAAAGCAAGACGAACAAAAGCATAATGAAAATGAAAATTCCAGAGAGCACCATCACCATCACC |
| Rpo21HTP Rev | TATTTGAGAAACTATATATAATGTAATAACGTCAAATACGTAAGGATGATATACTATATACGACTCACTATAGGG |
| Rpo21K1246R For | GTTATCTGGTCTGAAGACAACGATGAGAAGTTGATCATCCGTTGTCGTGTTGTTCGTCCAAGGTCACTAGATGCTGAG |

Table S4: DNA oligonucleotides used in this study.
