# Supplementary material for: RNA polymerase II stalling at pre-mRNA splice sites is enforced by ubiquitination of the catalytic subunit
Source: eLife. 2017 Oct 13;6:e27082. doi: 10.7554/eLife.27082 (PMC5673307; doi:10.7554/eLife.27082)
Supplement: Transparent reporting form [file elife-27082-transrepform.docx]

***eLife’s* transparent reporting form**

We encourage authors to provide detailed information *within their submission* to facilitate the interpretation and replication of experiments. If you have any questions, please contact us: [editorial@elifesciences.org](mailto:editorial@elifesciences.org).

**Sample-size estimation**

- You should state whether an appropriate sample size was computed when the study was being designed
- You should state the statistical method of sample size computation and any required assumptions
- If no explicit power analysis was used, you should describe how you decided what sample (replicate) size (number) to use

Please outline where this information can be found within the submission (e.g., page numbers or figure legends), or explain why this information doesn’t apply to your submission:

N/A

**Replicates**

- You should report how often each experiment was performed
- You should include a definition of biological versus technical replication
- The data obtained should be provided and sufficient information should be provided to indicate the number of independent biological and/or technical replicates
- If you encountered any outliers, you should describe how these were handled
- Criteria for exclusion/inclusion of data should be clearly stated
- High-throughput sequence data should be uploaded before submission, with a private link for reviewers provided (these are available from both GEO and ArrayExpress)

Please outline where this information can be found within the submission (e.g., page numbers or figure legends), or explain why this information doesn’t apply to your submission:

**CRAC experiments**

Datasets accessible through GEO, reviewer link:

<https://www.ncbi.nlm.nih.gov/geo/query/acc.cgi?token=gdufwymgpnmlxmh&acc=GSE94944>

Bre5-HTP, 2 reps

Bre5-HTP, ubp3∆ 2 reps

Bre5-HTP, rps5-3, 1 rep at permissive temperature,

Bre5-HTP, rps5-3, 1 rep at non permissive temperature (37°C)

Bre5-HTP, 1 rep at non permissive temperature (37°C)

BY4741 (untagged strain), 1 rep

Rpo21-HTP total, 2 reps

Rpo21-HTP, bre5∆ total, 2 reps

Ub-Rpo21-HTP, 2 reps

Rpo21-HTP, bre5∆ GST, 2 reps

Rpo21-HTP GST, 2 reps

Ub-Rpo21-HTP bre5∆, 2 reps

Rpo21 K1246R-HTP, 2 reps

**RNAseq experiments**

Datasets accessible through GEO, reviewer link:

<https://www.ncbi.nlm.nih.gov/geo/query/acc.cgi?token=gdufwymgpnmlxmh&acc=GSE94944>

Rpo21 K1246R-HTP, 4 reps poly(A)+

Rpo21-HTP, 2 reps poly(A)+

Rpo21 K1246R-HTP, 4 reps ribominus

Rpo21-HTP, 2 reps ribominus

**Figure 1B.** Data from triplicate experiments with standard deviation.

**Figure 1C.** Data from triplicate experiments

**Figure 1 D-H**. Metagene analysis. 2 independent experiments were performed

binding across protein coding transcripts, 5171 genes.

binding across intron containing transcripts, 288 genes.

**Figure 1 S1 B-G**. Metagene analysis.

binding across protein coding transcripts, 5171 genes.

binding across intron containing transcripts, 288 genes.

B-D. 1 experiment is shown for Bre5-HTP (another one was shown in Figure 1 D-H). 2 independent experiments are shown for Bre5-HTP, ubp3∆.

E-F. 1 experiment is shown for Bre5-HTP and Bre5-HTP, rsp5-3.

**Figure 1 S2.** Data from triplicate experiments.

**Figure 2** **B-G**. These experiments were done with three biological replicates. The qualitative results were consistent between biological replicates, however, slight differences in induction kinetics makes it difficult to combine these for statistical analyses. The graphs therefore show single experiments with technical replicates.

**Figure 2 S1.** As figure 2

**Figure 3. A** and **C** The RT-qPCR analysis resulted from 3 biological replicates with standard deviation shown.

**B** The in-vitro RNA-binding assay was done once with all the RRM mutants showing loss of binding.

**Figure 4**

**A** and **B** were only successfully done once due to technical difficulties with the anti-ubiquitin antibodies.

**C.** The mass spec was only done once as a qualitative experiment but the ubiquitinated residue was identified in 3 independent samples.

**D.** Two independent experiments were performed for each sample.

**Figure 5 D-F**. Metagene analysis.

An average of 3 independent experiments is shown for each dataset.

binding across protein coding transcripts, 5171 genes.

binding across intron containing transcripts, 288 genes.

**Figure 5 S1**.

**A.** was done once.

**B.** Number of reads for 1 experiment each.

**C-D**. The average of 3 independent experiments is shown for Ub-Rpo21 and for Ub-Rpo21, bre5∆. The standard error for each position is shown in different panels for each experiment.

binding across protein coding transcripts, 5171 genes.

binding across intron containing transcripts, 288 genes.

**Figure 6 A-C**. Metagene analysis.

An average of 3 independent experiments is shown for Ub-Rpo21, bre5∆.

An average of 2 independent experiments is shown for Rpo21K1246R.

binding across protein coding transcripts, 5171 genes.

binding across intron containing transcripts, 288 genes.

**Figure 6 D.** The RT-qPCR analysis resulted from 3 biological replicates with standard error shown

**Figure 6 E-J**. Splicing evaluation in RNAseq datasets.

**E.** 101 intron-containing mRNAs have been analyzed. Those represents transcripts with sufficient coverage. 4 Rpo21K1246R-HTP and 2 Rpo21-HTP replicates were used.

**F.** 296 intron-containing mRNAs were analyzed. 101 genes with high coverage are highlighted.

**G.** 85 intron-containing mRNAs with sufficient coverage were analyzed. 4 Rpo21K1246R-HTP and 2 Rpo21-HTP replicates were used.

**H.** 100 intron-containing mRNAs were analyzed. Those represents transcripts with sufficient coverage. 4 Rpo21K1246R-HTP and 2 Rpo21-HTP replicates were used.

**I.** 296 intron-containing mRNAs were analyzed. 100 genes with high coverage are highlighted 4 Rpo21K1246R-HTP and 2 Rpo21-HTP replicates were used.

**J.** 49 intron-containing mRNAs with sufficient coverage were analyzed. 4 Rpo21K1246R-HTP and 2 Rpo21-HTP replicates were used.

**Figure6 S1**. Splicing evaluation in RNAseq datasets. Graphs show pairwise comparisons of single datasets.

4 Rpo21K1246R-HTP and 2 Rpo21-HTP replicates were used.

**Statistical reporting**

- Statistical analysis methods should be described and justified
- Raw data should be presented in figures whenever informative to do so (typically when N per group is less than 10)
- For each experiment, you should identify the statistical tests used, exact values of N, definitions of center, methods of multiple test correction, and dispersion and precision measures (e.g., mean, median, SD, SEM, confidence intervals; and, for the major substantive results, a measure of effect size (e.g., Pearson's r, Cohen's d)
- Report exact p-values wherever possible alongside the summary statistics and 95% confidence intervals. These should be reported for all key questions and not only when the p-value is less than 0.05.

N/A

Please outline where this information can be found within the submission (e.g., page numbers or figure legends), or explain why this information doesn’t apply to your submission:

(For large datasets, or papers with a very large number of statistical tests, you may upload a single table file with tests, Ns, etc., with reference to page numbers in the manuscript.)

**Additional data files (“source data”)**

- We encourage you to upload relevant additional data files, such as numerical data that are represented as a graph in a figure, or as a summary table
- Where provided, these should be in the most useful format, and they can be uploaded as “Source data” files linked to a main figure or table
- Include model definition files including the full list of parameters used
- Include code used for data analysis (e.g., R, MatLab)
- Avoid stating that data files are “available upon request”

Please indicate the figures or tables for which source data files have been provided:

Source data for all figures are provided in GEO link.
